# Effect of Evolocumab on Grafts Post-Coronary Artery Bypass Surgery: Post-Hoc Analysis of Newton-CABG CardioLink-5

**DOI:** 10.1016/j.jacadv.2026.103162

**Published:** 2026-08-19

**Authors:** G.B. John Mancini, Craig Kamimura, Michael Szarek, Eunice Yeoh, Arnold Ryomoto, Hwee Teoh, Adrian Quan, Randi Elituv, Meena Verma, Elizabeth Misner, Tarit Saha, Bobby Yanagawa, Peter Jüni, Michael J. Koren, Stephen J. Nicholls, Deepak L. Bhatt, Lawrence A. Leiter, C. David Mazer, Subodh Verma

**Affiliations:** a Cardiovascular Imaging Core Laboratory, Centre for Cardiovascular Innovation, Dilawri Cardiovascular Institute, Division of Cardiology, Department of Medicine, University of British Columbia, Vancouver, British Columbia, Canada; b Division of Cardiology, University of Colorado School of Medicine, Aurora, Colorado, USA; c CPC Clinical Research, University of Colorado School of Medicine, Aurora, Colorado, USA; d Mount Sinai Fuster Heart Hospital, Icahn School of Medicine at Mount Sinai, New York, New York, USA; e Division of Cardiac Surgery, St Michael’s Hospital-Unity Health Toronto, Toronto, Ontario, Canada; f Division of Endocrinology and Metabolism, St Michael’s Hospital-Unity Health Toronto, Toronto, Ontario, Canada; g North York Diagnostic and Cardiac Centre, Toronto, Ontario, Canada; h Royal College of Surgeons in Ireland, Dublin, Ireland; i Department of Anesthesiology and Perioperative Medicine, Kingston Health Sciences Centre, Kingston, Ontario, Canada; j Department of Anesthesiology and Perioperative Medicine, Queen's University, Kingston, Ontario, Canada; k Department of Surgery, University of Toronto, Toronto, Ontario, Canada; l Department of Pharmacology and Toxicology, University of Toronto, Toronto, Ontario, Canada; m Institute of Health Policy, Management, and Evaluation, University of Toronto, Toronto, Ontario, Canada; n Clinical Trial Service Unit and Epidemiological Studies Unit (CTSU), Nuffield Department of Population Health, University of Oxford, Oxford, Oxfordshire, United Kingdom; o Jacksonville Center for Clinical Research, Flourish Research Group, Jacksonville, Florida, USA; p Victorian Heart Institute, Monash University, Melbourne, Victoria, Australia; q Department of Medicine, University of Toronto, Toronto, Ontario, Canada; r Department of Nutritional Sciences, University of Toronto, Toronto, Ontario, Canada; s Department of Anesthesia, St Michael’s Hospital-Unity Health Toronto, Toronto, Ontario, Canada; t Department of Anesthesiology and Pain Medicine, University of Toronto, Toronto, Ontario, Canada; u Department of Physiology, University of Toronto, Toronto, Ontario, Canada

**Keywords:** evolocumab, graft failure, internal mammary artery graft, low attenuation plaque, radial artery graft, saphenous vein graft

## Abstract

**Background:**

Graft failure remains a significant clinical problem.

**Objectives:**

This study aimed to evaluate effects of evolocumab on graft failure, stenosis severity, stenosis types, and plaque burden in saphenous vein grafts (SVGs), internal mammary artery (IMA), and radial artery grafts using coronary computed tomography angiography (CCTA).

**Methods:**

Analysis of the randomized NEWTON-CABG CardioLink-5 trial was undertaken in evaluable grafts 24 months postsurgery. Graft stenosis was graded normal: <20%, moderate: 20% to <50%, or graft failure: 50% to 100%. Stenoses ≥20% were classified: positive remodeling, >25% low-attenuation plaque (LAP), or spotty calcification. Plaque burden (calcified, mixed, or LAP) was measured.

**Results:**

We studied 1,275 SVGs, 514 IMA grafts, and 39 radial artery grafts. There were no differences between treatment groups for graft failure in any graft type. Evolocumab had a lower percentage of moderate lesions among SVGs (11.5% vs 19.3%, *P* < 0.001), a similar nonsignificant trend in IMA grafts (3.5% vs 5.9%), and fewer focal stenoses ≥20% showing positive remodeling, >25% LAP, or spotty calcification. SVGs accumulated more plaque burden but with greater percentage calcified (*P* < 0.002) and mixed plaque (*P* < 0.001) and less percentage LAP (*P* < 0.001) than IMA grafts.

**Conclusions:**

Evolocumab did not improve early graft failure, but exploratory results suggest that it may diminish moderate stenoses in SVGs and stenoses with unfavorable features. SVGs accumulated more plaque burden than IMA grafts but with less percentage LAP, possibly due to the early arterialization process involving smooth muscle cell proliferation, neointimal hyperplasia, and extracellular matrix expansion affecting only the SVGs.

Coronary artery bypass graft (CABG) surgery is one of the most frequently performed procedures worldwide. But up to 20% of saphenous vein grafts (SVGs) become totally occluded within the first year after surgery, and approximately 40% are 100% occluded by 10 years, with an additional 50% exhibiting advanced atherosclerotic changes.^1^, ^2^, ^3^ Internal mammary artery (IMA) grafts fare much better than SVGs with early failure rates of between 3% and 9%.^4^^,^^5^ Radial artery (RA) graft failure rates are reportedly between 10% and 25%.^4^, ^5^, ^6^, ^7^, ^8^, ^9^ Mitigation of these rates requires understanding of the underlying biology and targeted interventions.^10^

Low-density lipoprotein cholesterol (LDL-C) is a prognostic marker in CABG populations, and LDL-C lowering has been shown to decrease clinical events.^11^^,^^12^ But effects on graft patency have been less clear. Notably, in the ACTIVE (Aggressive Cholesterol Therapy to Inhibit Vein Graft Events) trial, there was no significant difference between high- and low-intensity statin therapy in SVG occlusion at 1 year, despite a 25% differential in achieved LDL-C levels.^13^ Proprotein convertase subtilisin/kexin type 9 (PCSK9) inhibitors, such as evolocumab, are highly effective LDL-C-lowering agents that significantly reduce atherogenic lipoprotein levels and consistently decrease major adverse cardiovascular events in patients with established atherosclerotic cardiovascular disease.^14^^,^^15^ In well-advanced plaques, evolocumab has been shown to promote coronary plaque regression, stabilize high-risk plaques, and reduce necrotic core volume in carotid atherosclerosis.^16^, ^17^, ^18^

The NEWTON-CABG CardioLink-5 trial assessed the impact of evolocumab initiated in the early postoperative period (21 days or less post-CABG surgery) and on a background of moderate- to high-intensity statin therapy on SVG patency 2 years following CABG surgery. It demonstrated a neutral result.^19^ The primary purpose of this analysis is to assess and compare the effects of evolocumab on graft failure of SVG, IMA, and RA grafts. Secondarily, we assess degree of stenosis, type of focal lesions (low attenuating plaques [LAP], positive remodeling, and development of spotty calcification), and the components of overall plaque burden affecting the different graft types.

## Methods

The NEWTON-CABG CardioLink-5 trial is registered with ClinicalTrials.gov (NCT03900026). The trial protocol was approved by the applicable independent research ethics or Institutional Review Boards in each country and site.^19^ Subjects provided informed, written consent through their local Institutional Review Board for enrollment into the original trial. Graft patency was assessed by coronary computed tomography angiography (CCTA) at the 24-month (±2 months) visits. All CCTA images were processed and interpreted by the Cardiovascular Imaging Research Core Laboratory at the University of British Columbia. The standard operating procedure for the CCTA process is found in the Supplemental Materials. Readers were blinded to treatment allocation. The image analyses were performed by 3 core lab technicians, each with >10 years of core lab experience assessing CCTA. All image analyses were performed in a double-blind fashion, and data lock occurred prior to analyses. Quantitative plaque characteristics were evaluated according to previously published standard operating procedures using Vitrea Advanced Visualization, CTA module, version 7.15.^20^, ^21^, ^22^ The most severe diameter stenosis was recorded for each evaluable graft, and the graft was categorized as normal (<20% stenosis), moderately stenotic (20% to 49% stenosis), or graft failure (≥50% to 100%). In nonoccluded grafts, focal lesions ≥20% were characterized by the presence of spotty calcification, the presence of vascular remodeling (ratio of area at site of maximal narrowing to reference area ≥1.1), or presence of >25% LAP (<30 HU) of the focal lesion volume.^23^, ^24^, ^25^ Nonoccluded grafts were also assessed along their entire lengths, and 3 plaque categories were measured using fixed HU as previously described.^21^^,^^22^^,^^26^, ^27^, ^28^: LAP (<30 HU), mixed plaque (30-350 HU), and calcified plaque (>350 HU). No imputation was used for subjects without 24-month follow-up CCTA or for measures of plaque when grafts were 100% occluded or technically nonevaluable due to inadequate imaging quality.

### Statistical analysis

Analyses were performed on all randomized participants who received at least one dose of the investigational product and had at least one graft evaluable for plaque volume at 24 months. Continuous variables are expressed as mean (95% CI), while categorical variables are expressed as counts and percentages.

Endpoints with 2 categories were analyzed by logistic regression models, with results summarized by ORs with likelihood ratio-based 95% CIs and *P* values. For endpoints with 3 ordered categories (ie, degree of graft stenosis), ordinal logistic regression models were applied with cumulative logit link functions. Since graft was the unit of analysis and endpoints included one or more assessments per participant, a generalized estimating equation approach with an exchangeable correlation structure was applied to account for within-participant correlation. For analyses involving treatment group comparisons, randomized treatment assignment was the only predictor in the models. For analyses involving comparisons of graft types, ORs, 95% CIs, and *P* values reflected adjustment for randomized treatment.

Absolute and percent plaque volumes were analyzed by linear regression models with randomized treatment assignment as the only predictor. Absolute volume was divided by the number of grafts that were evaluated per participant, across the different types or for each specific type. Percent volume was quantified using normalization by vessel volume. For analyses involving comparisons of graft types, means, 95% CIs, and *P* values reflected adjustment for randomized treatment.

Two-sided *P* values <0.05 were considered statistically significant, with no adjustment for multiplicity. Analyses were performed in SAS version 9.4.

## Results

The previously published main trial and the current analysis are based on a modified intention-to-treat basis. The 2 treatment arms were balanced with respect to demographics, baseline LDL-C, and background cardiovascular medications. It demonstrated neutral results for SVG failure (50% to 100% stenosis) between the placebo- and evolocumab-treated arms.^19^ For this analysis, there were 273 subjects in the placebo group with evaluable stenosis in at least one graft (622 SVGs, 254 IMA grafts, and 24 RA grafts) that were evaluable by 24-month follow-up CCTA scans. There were 277 subjects in the evolocumab group with evaluable stenosis in at least one graft (653 SVGs, 260 IMA grafts, and 15 RA grafts) (Supplemental Figure 1). The baseline characteristics are shown in Supplemental Table 1, and there were no imbalances.

### Stenosis severity category by treatment and by type of graft

**Figure 1:**
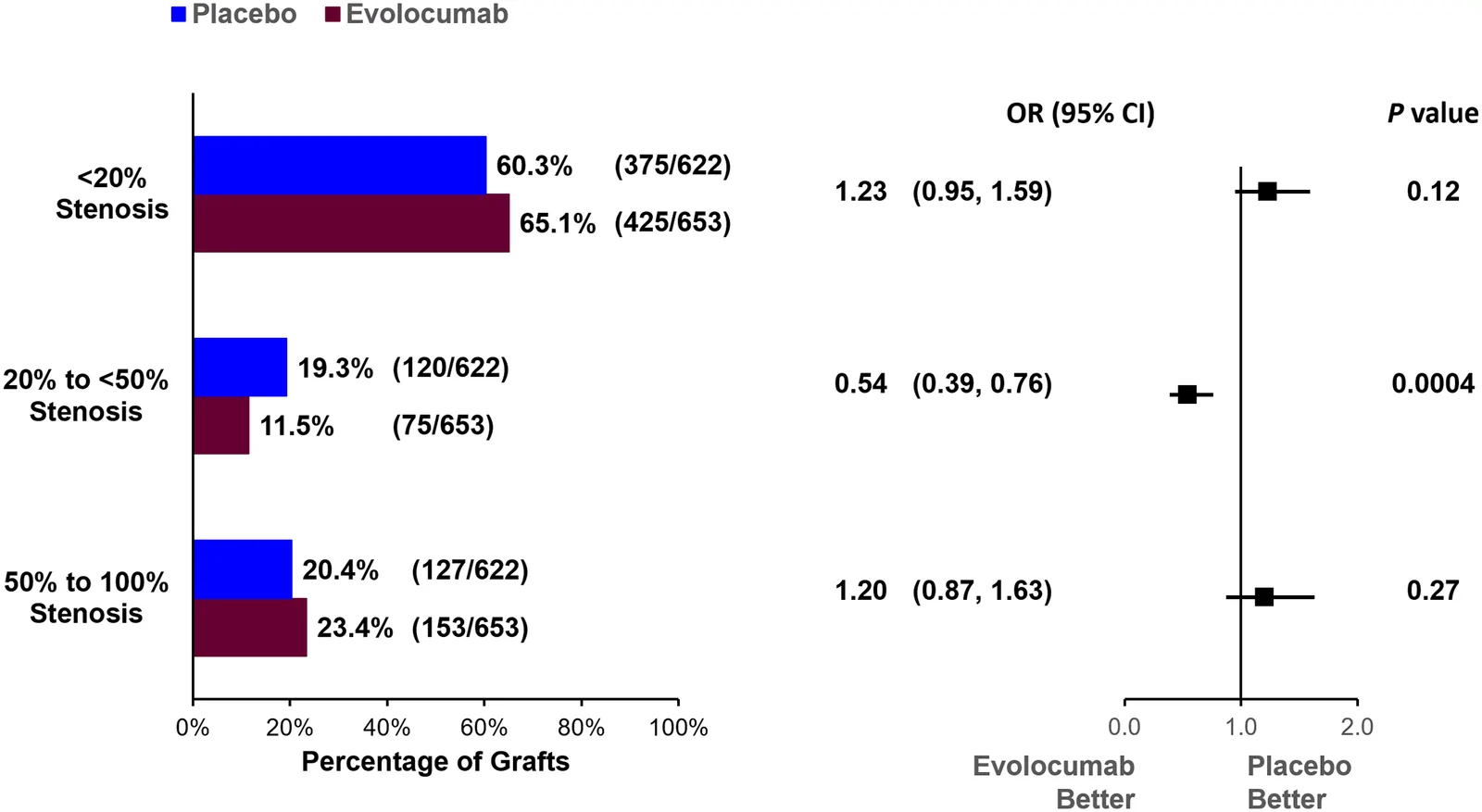
Effects of Evolocumab and Placebo on Stenosis Severity Evolocumab was associated with a 46% lower OR (OR [95% CI] = 0.54 [0.39-0.76]; *P* < 0.0004) in the formation of moderate (20 to < 50%) stenoses in saphenous vein bypass grafts. ORs were obtained from logistic regression models.

There were no overall differences in the effects of evolocumab across the 3 categories of focal stenosis severity: OR: 0.90; 95% CI: 0.69-1.16; *P* = 0.43 for SVGs; OR: 0.98; 95% CI: 0.64-1.50; *P* = 0.93 for IMA grafts; OR: 1.10; 95% CI: 0.28 to 4.36, *P* = 0.90 for RA grafts, including graft failure (≥50% stenosis). However, there was a greater proportion of moderate lesions (20 - <50%, 19.3% vs 11.5%, *P* < 0.001) in SVGs in the placebo- vs the evolocumab-treated group, respectively (Figure 1). A similar numerical trend was seen for moderate lesions in IMA grafts (5.9% in the placebo group vs 3.5% in the evolocumab group, *P* = 0.20) but not for RA grafts (Supplemental Figures 2 and 3).

**Figure 2:**
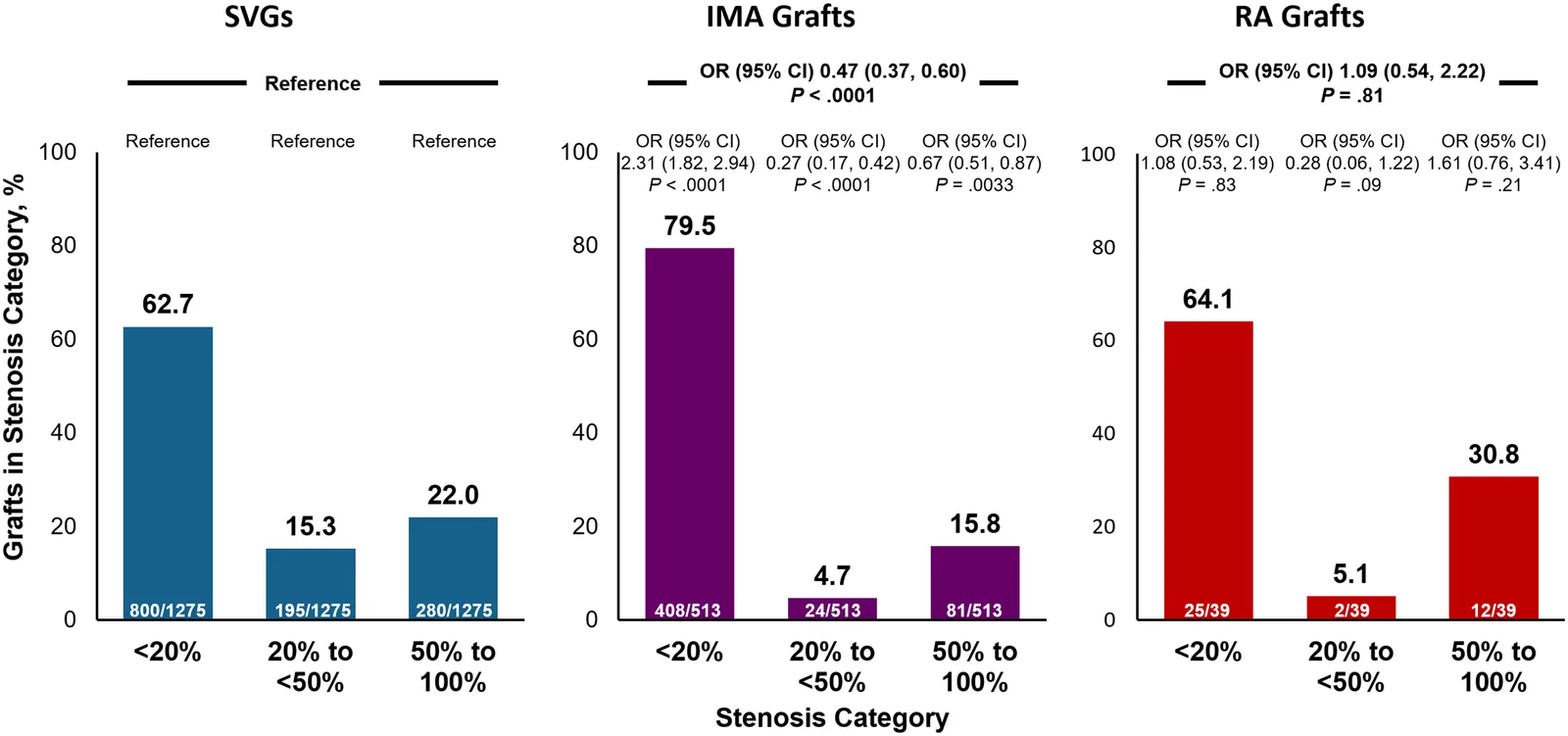
Stenosis in Saphenous Vein, Internal Mammary, and Radial Artery Grafts Using saphenous vein grafts as the comparator, internal mammary artery grafts were more likely to remain normal (<20% stenosis), less likely to fail (50% to 100% occlusion), and less likely to form moderate stenosis (20% to <50%). These differences were not seen between the saphenous vein graft and radial artery grafts. IMA = internal mammary artery; RA = radial artery; SVG = saphenous vein graft.

With respect to SVGs vs arterial grafts, performance according to the 3 stenosis severity categories was not different between SVGs and RA grafts. In contrast, IMA grafts showed a significantly greater propensity to remain normal (<20%; OR: 2.31, 95% CI: 1.82-2.94, *P* < 0.001), and with fewer moderate stenoses (20 - <50%; OR: 0.27, 95% CI: 0.17-0.42, *P* < 0.001) and fewer graft failures (50% to 100%; OR: 0.67; 95% CI: 0.51-0.87; *P* = 0.003). These results are summarized in Figure 2.

### Characteristics of focal lesions by treatment and by type of graft

**Figure 3:**
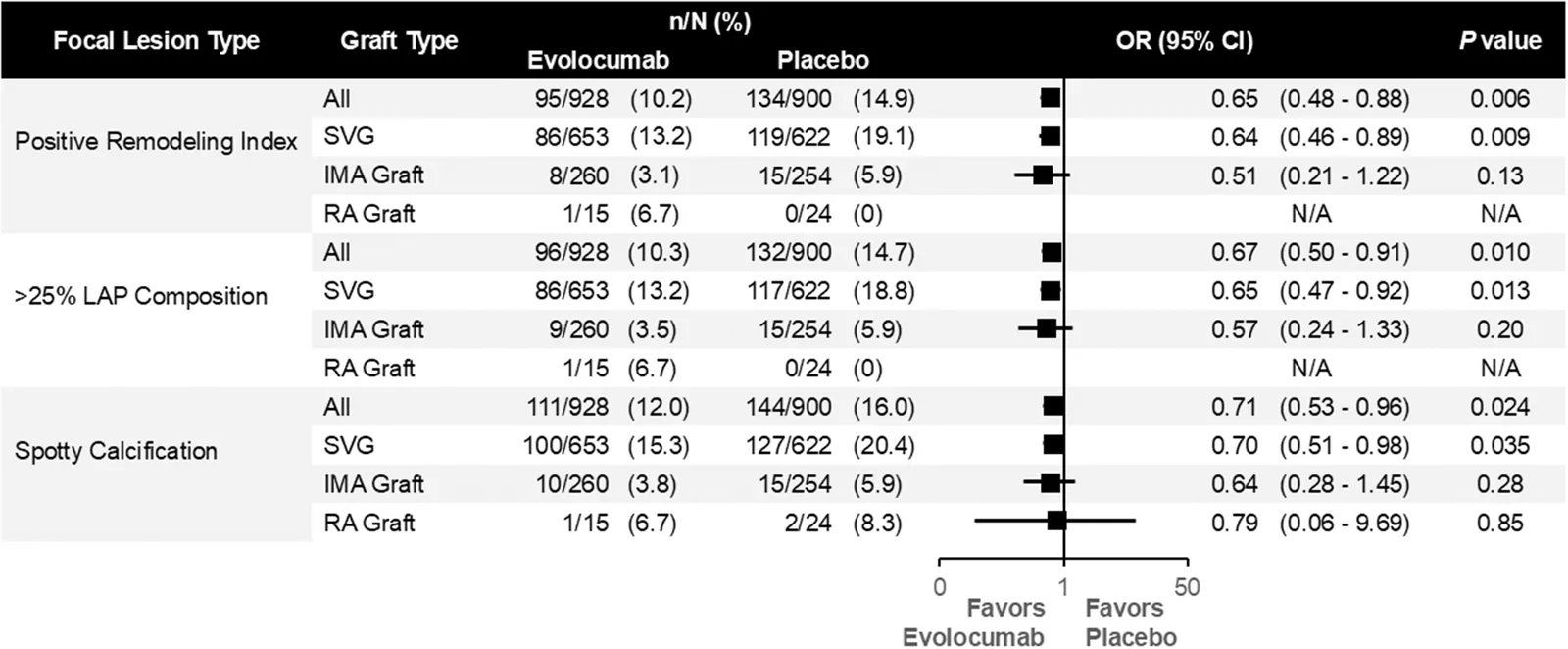
Effects of Evolocumab on Characteristics of Focal Lesions Evolocumab was associated with significant reduction in development of focal lesions with unfavorable characteristics such as positive remodeling, >25% low attenuation plaque composition and spotty calcification. These results were largely influenced by the significant individual analyses of the saphenous vein grafts. LAP = low-attenuation plaque; N/A = not applicable; other abbreviations as in Figure 2.

Evolocumab was associated with a significant reduction in the number of focal lesions >20% in SVGs associated with positive remodeling (OR: 0.64; 95% CI: 0.46-0.89; *P* = 0.009), >25% LAP composition (OR: 0.65; 95% CI: 0.47-0.92; *P* = 0.013), or spotty calcification (OR: 0.70; 95% CI 0.51-0.98; *P* = 0.035). Despite trends in a similar direction, these differences were not statistically significant in IMA grafts (positive remodeling: OR: 0.51; 95% CI: 0.21-1.22; *P* = 0.13; >25% LAP composition: OR: 0.57; 95% CI: 0.24-1.33; *P* = 0.20; spotty calcification: OR: 0.64 (95% CI: 0.28-1.45, *P* = 0.28). There were too few instances in the RA grafts to allow statistical comparison. These results are summarized in Figure 3.

**Figure 4:**
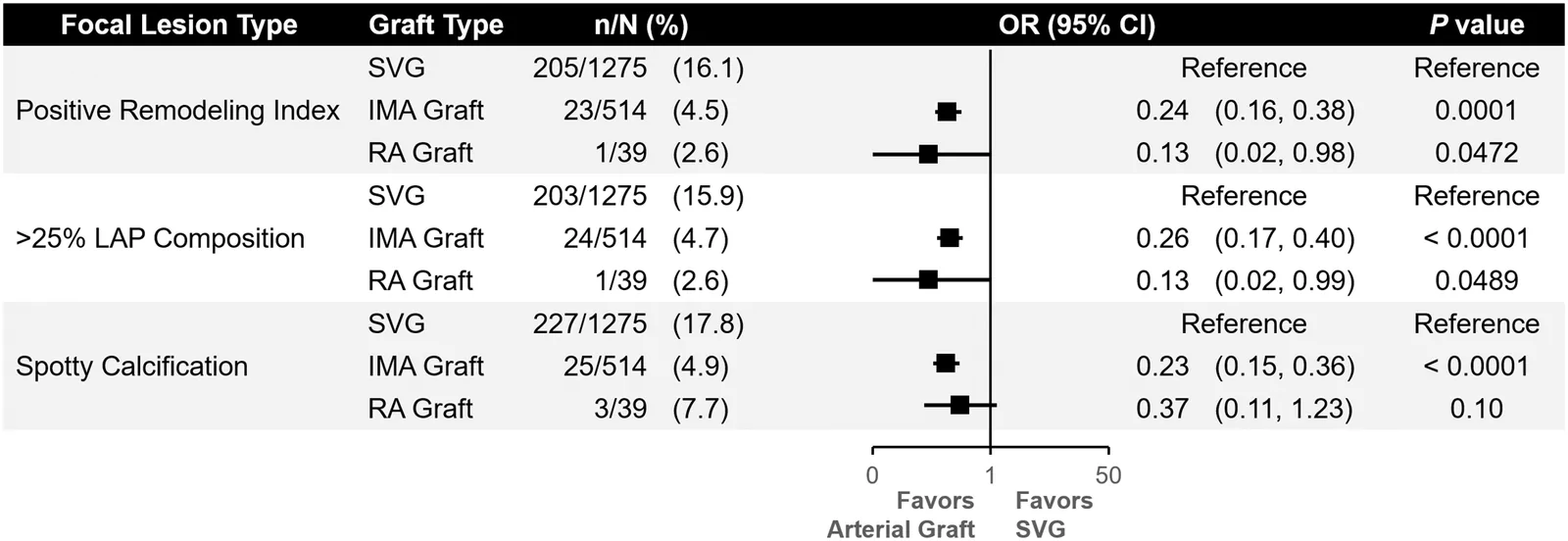
Characteristics of Focal Lesions in Arterial Grafts Compared With Saphenous Vein Grafts Internal mammary artery grafts showed significantly reduced ORs for the development of plaques with unfavorable characteristic as compared with saphenous vein grafts. Trends were similar in the radial artery grafts and statistically significant with respect to lesions with positive remodeling or >25% low-attenuation plaque composition. Abbreviations as in Figures 2 and 3.

There were significantly fewer focal lesions in the IMA grafts compared with SVGs: positively remodeled lesions (OR: 0.24; 95% CI: 0.16-0.38; *P* < 0.001), lesions with >25% LAP (OR: 0.26; 95% CI: 0.17-0.40; *P* < 0.001), and lesions with spotty calcification (OR: 0.23; 95% CI: 0.15-0.36; *P* < 0.001). There were similar trends in the RA grafts (positive remodeling: OR: 0.13; 95% CI: 0.02-0.98, *P =* 0.047; >25% LAP lesions: OR: 0.13; 95% CI: 0.02-0.99; *P* = 0.049; spotty calcification: OR: 0.37; 95% CI: 0.11-1.23; *P* = 0.10) (Figure 4).

#### Plaque volumes by treatment and by type of graft

Over the entire length of the conduits, there were no statistically significant differences between placebo- and evolocumab-treated subjects based on either the absolute or percentage accumulation of LAP or calcified plaque among any of the graft types. There was a nominal reduction in the percentage of mixed plaque in RA grafts in the evolocumab-vs placebo-treated subjects (40.5% vs 44.9%, respectively, *P* = 0.03) (Supplemental Table 2).

**Figure 5:**
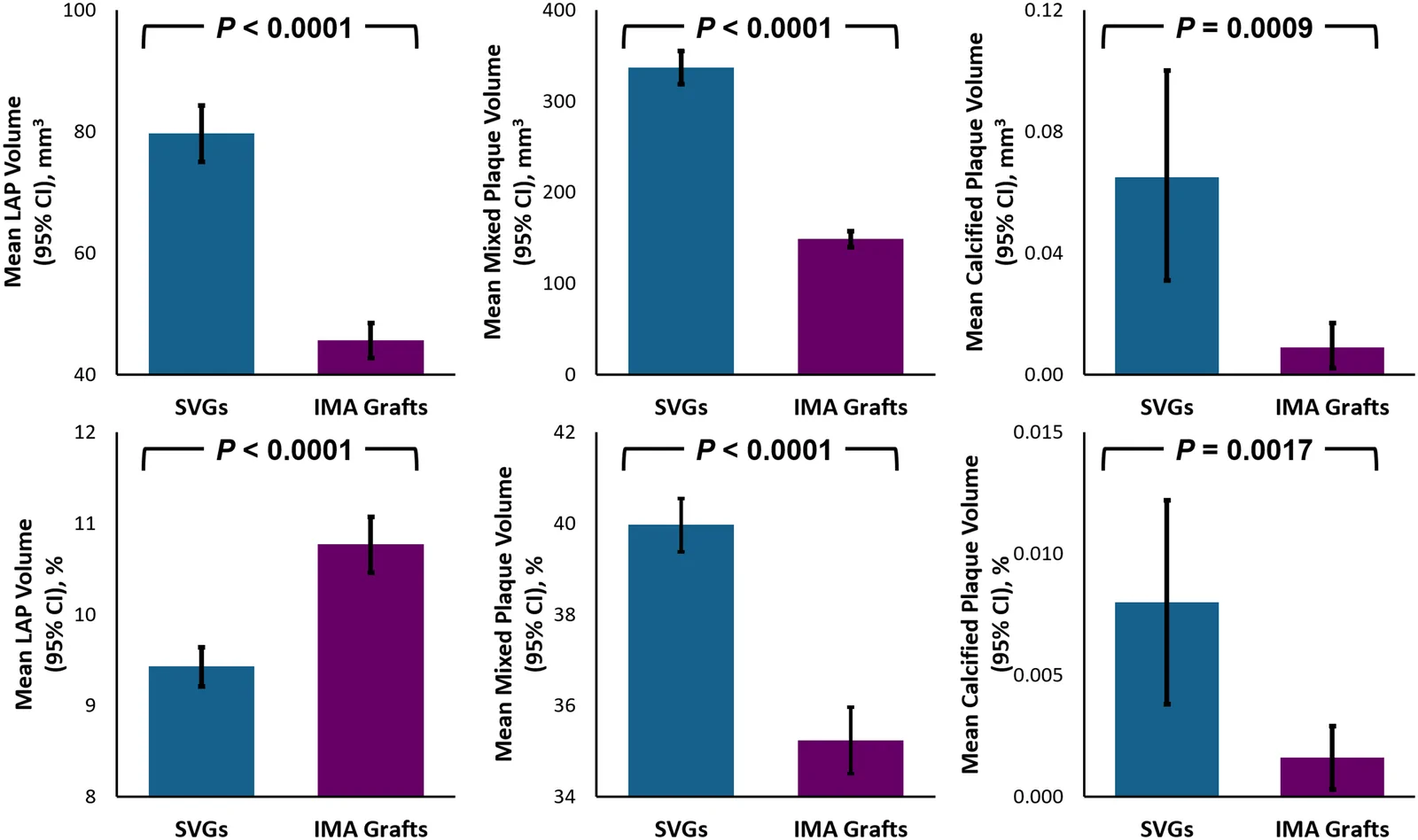
Plaque Characteristics in Saphenous Vein and Internal Mammary Artery Grafts Upper panels show absolute volumes and lower panels show percentage volumes. Accumulation of plaque of any nature was significantly greater in saphenous vein grafts than in internal mammary artery grafts. However, the percentage of the different components was qualitatively different (lower panels). Plaque in the saphenous vein grafts contained proportionally more mixed and calcified plaque and less low-attenuation plaque than the internal mammary artery grafts. Abbreviations as in Figures 2 and 3.

Because of the small number of RA grafts and the absence of calcification in RA grafts, more detailed analyses of overall plaque were performed only on the SVGs and IMA grafts and were adjusted for treatment allocation. The SVGs showed significantly more accumulation of plaque volume whether LAP, mixed, or calcified (Figure 5 upper panels). But the accumulated plaque differed in the proportion of its components. The SVG plaque was characterized by a significantly greater percentage of mixed and calcified plaque (*P* < 0.001 and *P* = 0.002, respectively), whereas the percent LAP volume was higher in IMA grafts than in the SVG (*P* < 0.001) (Figure 5 lower panels). Sample images are demonstrated in the Central Illustration.

**Central Illustration:**
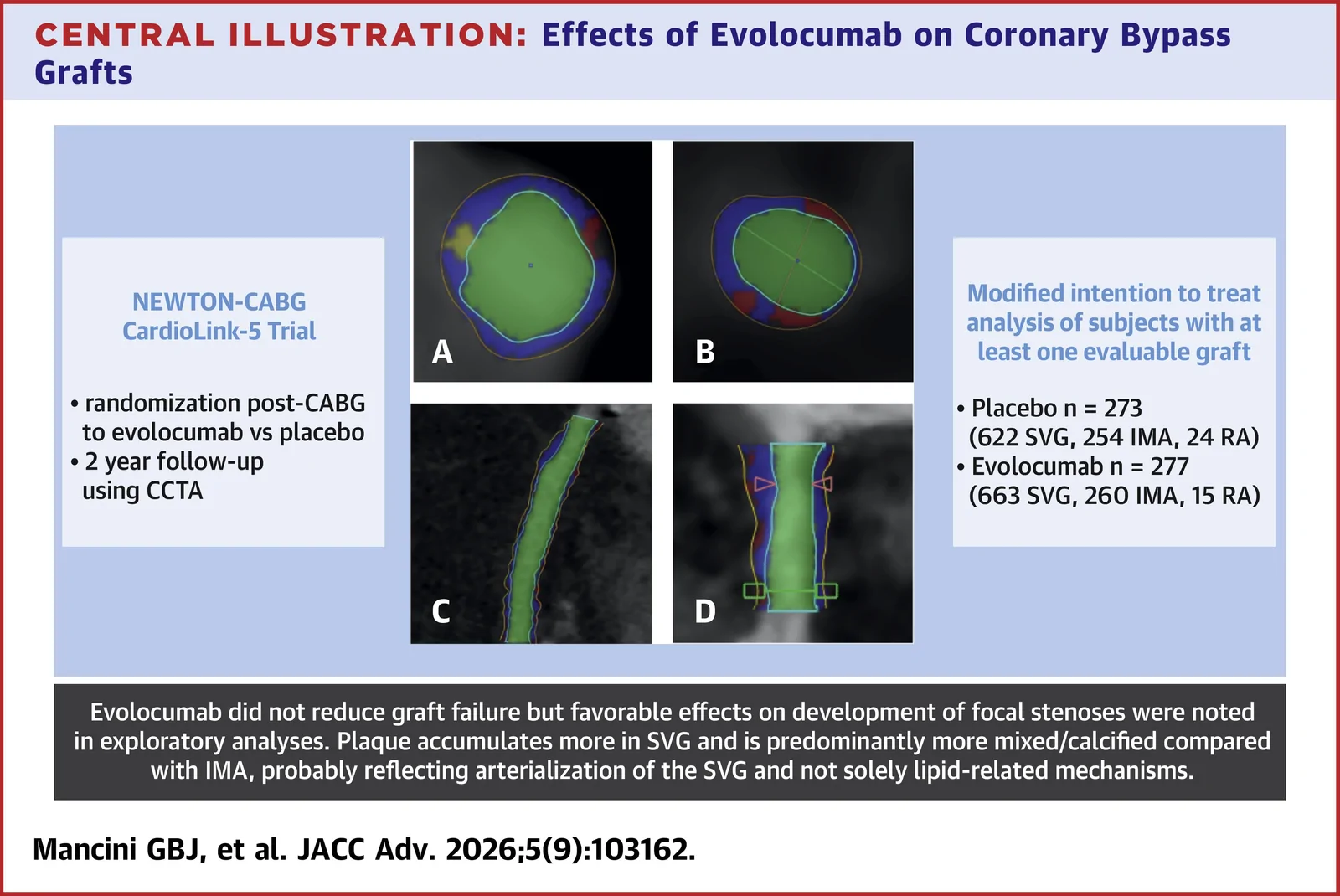
Effects of Evolocumab on Coronary Bypass Grafts Images are color coded: lumen, green; calcification, yellow; mixed plaque, blue; low attenuation plaque, red; external vessel wall, orange. (A) Cross section of a saphenous vein graft demonstrating calcification, mixed plaque, and low attenuation plaque. The low attenuation plaque area is 6.6%. (B) Cross section of a left internal mammary artery graft with low attenuation plaque of 31.4% and no calcium. (C) Longitudinal saphenous vein graft. The wall of the saphenous vein graft demonstrates mixed plaque and areas of low attenuation plaque. No calcium is present. A mild, focal stenosis (25%) is present in the proximal portion. (D) Longitudinal view of a saphenous vein stenosis where the external vessel wall at the site of maximal stenosis (orange outer line at red arrows) is larger than at the reference area (green rectangles) yielding a positive remodeling index of 1.47. CCTA = coronary computed tomography angiography; other abbreviations as in Figures 2 and 3.

## Discussion

Consistent with the main study results, this post hoc analysis demonstrated no statistically significant difference in the primary efficacy endpoint of 24-month graft failure (50% to 100% diameter stenosis) between the evolocumab and placebo groups for not only SVGs but also the IMA and RA grafts.^19^ However, evolocumab treatment was associated with significantly fewer moderate focal stenoses (20% to 49%) in SVGs with an approximate halving of this rate from 19.3% to 11.5%. In IMA grafts, the rates of moderate stenoses were substantially lower but with similar directionality (5.9% in the placebo group and only 3.5% in the evolocumab group). Finally, in nonoccluded grafts of any type, but largely driven by the SVGs, evolocumab was associated with a decreased number of focal lesions ≥20% showing unfavorable features (spotty calcification, positive remodeling, or >25% LAP).

Prior trials have examined whether early LDL-C intensification and other interventions can reduce the biological substrate that predisposes to subsequent graft disease.^13^^,^^29^, ^30^, ^31^ Collectively, these data support the notion that thrombosis and intimal hyperplasia, rather than early atherosclerosis, may drive early SVG failure and may not be amenable to further LDL-C reduction within this early time frame. While evolocumab resulted in the anticipated ∼60% relative reduction in LDL-C, the median baseline LDL-C level in participants was already relatively low at ∼1.9 mmol/L (73 mg/dL) at baseline and ∼1.6 mmol/L (62 mg/dL) in the placebo group at end of trial. The evolocumab-treated group had an end of trial LDL-C of ∼0.8 mmol/L (31 mg/dL) yielding an absolute difference of only 0.8 mmol/L (31 mg/dL), perhaps precluding detection of more profound effects of the intensive LDL-C lowering with evolocumab. Moreover, unique to the SVGs in this early postoperative phase is the abrupt, de novo exposure to arterial pressure that promotes intimal and smooth muscle cell hyperplasia, extracellular matrix expansion, and thrombotic processes, with traditional atherosclerosis typically manifesting later.^1^^,^^10^ Accordingly, the effects of lowering of LDL-C are intertwined with other mechanisms of remodeling. The LDL-C lowering itself may reduce oxidized LDL, maintain endothelial function, suppress signaling pathways, and reduce adhesion molecules and other factors that may favorably affect other remodeling processes mediated through smooth muscle cells and arterialization of the vein.^32^, ^33^, ^34^ It is conceivable that these factors explain why we do demonstrate subtle but potentially beneficial effects of evolocumab with respect to diminished formation of moderate lesions and lesions with unfavorable characteristics in the early post-bypass phase. The results are also consistent with a recent post hoc analysis demonstrating a relationship between on-treatment LDL-C and SVG patency.^35^ Whether these early effects, coupled with frank atherosclerosis attenuation over the longer term, would amount to improvement in SVG failure will require additional, extended trials.

This analysis of the NEWTON-CABG CardioLink-5 trial experience is the first to compare graft types using CCTA and plaque component measurements. SVGs accumulated not only more plaque than IMA grafts but, importantly, the plaque was proportionately more of a mixed and calcified nature compared with the quality of plaque in the IMA grafts, which showed a greater proportion of LAP. This unique finding further supports the concepts outlined above that arterialization of SVGs dominates more so in the early postoperative years by accumulation of smooth muscle cells and fibrotic tissues and less so through accumulation of LDL-C-driven LAP, the latter being more evident in the IMA grafts. Accordingly, it is conceivable that maintenance of very low levels of LDL-C and longer-term follow-up, past the arterialization phase of SVGs, might show improved performance of both SVG and arterial grafts.

Although this CCTA project is unique, the results are compatible with insights obtained in prior studies of grafts using intravascular ultrasound imaging and angioscopy. Findings include evidence of higher accumulation of plaque in SVG compared with arterial grafts, early wall thickening and adaptive remodeling of SVG predisposing to the development of atherosclerotic plaque that is predominantly of a fibrotic nature, and, several years after the operation, evidence of yellow atheromatous plaques by angioscopy.^36^, ^37^, ^38^, ^39^, ^40^

Prior clinical trials, such as ODYSSEY OUTCOMES (Evaluation of Cardiovascular Outcomes After an Acute Coronary Syndrome During Treatment With Alirocumab), have demonstrated that the clinical efficacy of LDL-C-lowering therapies is greater in people who have higher baseline LDL-C levels, where the absolute reduction is larger.^41^ Likewise, participants in the GLAGOV (GLobal Assessment of Plaque reGression With a PCSK9 antibOdy as Measured by intraVascular Ultrasound) trial that reported regression of well-established, mature atherosclerotic plaque with evolocumab treatment had a baseline LDL-C of ∼2.4 mmol/L (93 mg/dL), with evolocumab reducing LDL-C by an additional 59%, corresponding to an absolute reduction of approximately 1.4 mmol/L (54 mg/dL), a difference much larger than noted in NEWTON-CABG CardioLink-5.^18^ Additionally, it must be emphasized that NEWTON-CABG CardioLink-5 is distinct in measuring not the progression/regression of well-established plaques but the initiation of plaques in venous and arterial conduits free of atherosclerosis to begin with.

### Study limitations

Limitations of this study should be understood within the context of the complex mechanisms leading to graft failure beyond lipids: surgical technical issues (including competing flow and poor distal vessels), inflammatory and thrombotic mechanisms, arterialization specific to vein grafts and the temporal differences in peak effects of each of these mechanisms of graft failure.^10^ This trial was conducted during the COVID-19 pandemic, which necessitated protocol modifications. Originally, baseline CCTA prior to randomization was planned to exclude technical failures, but logistical constraints imposed during the pandemic precluded this. Consequently, early graft failure due to technical issues—likely unresponsive to LDL-C modulation—cannot be excluded and may have diluted the treatment effect, although such failures were presumed to be evenly distributed between the 2 study arms. Second, the follow-up duration was limited to 24 months, which restricts our ability to comment on longer-term graft patency, so it remains uncertain if this early initiation of PCSK9 inhibitor therapy may yield more favorable results during extended follow-up. Third, detailed plaque morphology could not be measured in totally occluded grafts, thereby necessitating the modified intention-to-treat methodology. Fourth, analyses involving right IMA and RA grafts were markedly impeded by the low rate of utilization of these conduits. Fifth, the results are exploratory and not prespecified. Sixth, because of the exploratory nature of this project, data were not adjusted for multiplicity and should be interpreted in this context. Seventh, given the known altered rates of progression/regression in bypassed segments, there was no a priori plan to analyze native vessel segments. Finally, the trial was not powered for clinical events and so the neutral effects on overall graft failure in any of the graft types should not detract from the established cardiovascular benefits of longer term, intensive LDL-C lowering with evolocumab in individuals who have previously undergone CABG surgery.^11^^,^^14^^,^^42^^,^^43^ Secondary prevention therapies that include PCSK9 inhibitors,^11^ icosapent ethyl,^44^ sodium glucose co-transporter-2 inhibitors,^45^ and glucagon-like peptide-1 receptor agonists^46^ remain important in this population.^47^^,^^48^

## Conclusions

Among individuals undergoing CABG surgery, the addition of evolocumab to moderate- to high-intensity statin therapy within a median of 21 days post-CABG surgery and with a baseline median LDL-C of 1.85 mmol/L (72 mg/dL) did not improve 24-month failure of SVG, IMA, or RA grafts. The rate of moderate (20%-49%) focal stenoses and the formation of focal stenoses with unfavorable features (positive remodeling, spotty calcification, or > 25% LAP) was significantly reduced in SVGs in the evolocumab-treated arm. Importantly, plaque accumulation was significantly greater in SVG vs IMA grafts and of a different quality. The increased proportion of LAP in IMA grafts compared with SVG provides direct evidence that early plaque formation in arterial grafts is predominantly sensitive to lipid-related mechanisms, whereas plaque formation in SVG is more complex and reflects both arterialization- and lipid-related processes. Addressing the challenge of early and late graft failure will require therapies tailored to address these fundamental differences.

Perspectives **COMPETENCY IN PATIENT CARE:** In the post-CABG patient, although the rates of graft failure were similar in the short term with LDL-C in the range of <1.6 mmol/L (<62 mg/dL), maintenance of optimal LDL-C levels has proven clinical benefit. Whether sustained and very low levels of LDL-C will improve graft failure rates beyond 2 years post-CABG is unknown. These results imply that development of atherosclerosis and arteriosclerosis early after surgery through LDL-C lowering is complicated in SVGs due to the additional process of arterialization.

**TRANSLATIONAL OUTLOOK:** Early SVG graft failure is multifactorial, including not only lipid-related mechanisms but also mechanisms underlying arterialization. Targeted therapies for these processes, beyond lipid lowering, require identification and clinical application in future trials.

## Funding support and author disclosures

This investigator-initiated study was funded by Amgen Canada. The trial funder had no role in trial design, data collection, data analysis, data interpretation, or writing of the report. The decision to submit and where were at the discretion of the authors. Dr Mancini has received research support from, speaking honoraria, and/or acted as an advisor to Amgen, AstraZeneca, Boehringer Ingelheim, Eli Lilly, GSK, HLS Therapeutics, Merck, Novartis, Novo Nordisk, Esperion, and Ultragenyx. Dr Szarek has received salary support from CPC, a nonprofit academic research organization affiliated with the University of Colorado, that receives or has received research grant/consulting funding between July 2021 and July 2025 from the following organizations: 35Pharma, Inc, Abbott Laboratories, Agios Pharmaceuticals, Inc, Alexion Pharma Godo Kaisha, American Heart Association, American Journal Managed Care, Amgen Inc, Amgen USA, Inc, Anthos Therapeutics, Inc, Arrowhead Pharmaceuticals, AstraZeneca Pharma India, AstraZeneca Pharmaceuticals LP, AstraZeneca UK Ltd, Autonomy Bio, Inc, Bayer, Bayer Aktiengesellschaft, Beth Israel Deaconess Medical Center, Better Therapeutics, Boston Clinical Research Institute, LLC, Bristol Myers Squibb, Cleerly, Inc, Clergy United for the Transformation of Sandtown, Colorado Dept of Public Health and Environment, Congress Inc, Cook Regentec LLC, Eidos Therapeutics, Inc, EluraBio, Inc, Esperion Therapeutics, Inc, Faraday Pharmaceuticals, Inc, Gasherbrum Bio Inc, Insmed, IsomAb Limited, JanOne Biotech Holdings, Inc, Janssen Global Services, Janssen Pharmaceuticals, Inc, Janssen Scientific Affairs LLC, Las Animas and Huerfano Counties, District Health Dept, Lexicon Pharmaceuticals, Inc, Lilly USA, LLC, Medison Pharma, key, Inc, Merck Sharp & Dohme Corp., Nectero Medical, Inc, NewAmsterdam Pharma, Novartis Pharmaceuticals Corporation, Novo Nordisk Inc, Pfizer, Piper Sandler & Co, PPD Development, L.P., Prothena Biosciences Limited, Regeneron, Regents of the University of Colorado (aka UCD), Sanifit Therapeutics S.A., Sanofi, Silence Therapeutics PLC, Stanford University, Stealth BioTherapeutics Inc, The Brigham and Women's Hospital, Thrombosis Research Institute, Tourmaline Bio, Inc, University of Colorado, University of Pittsburgh, VarmX, Verve Therapeutics, WraSer, LLC; and has served as a consultant or research support (or both) from Amarin, Lexicon, NewAmsterdam, Novartis, Regeneron, Sanofi, Silence, and Tourmaline. Dr Teoh has received personal fees from the Canadian Medical and Surgical Knowledge Translation Research Group and LMC Healthcare. Dr Yanagawa has received speaker fees from Edwards Lifesciences. Dr Koren is an employee of a clinical research company supported by grants from multiple manufacturers of lipid and other therapeutics. Dr Nicholls has received grant/research support from Amgen, Anthera, AstraZeneca, Cerenis, Cyclarity, Eli Lilly, Esperion, InfraReDx, NewAmsterdam Pharma, Novartis, Resverlogix, Roche, Sanofi-Regeneron, The Medicines Company, and LipoScience; and was a consultant for Abcentra, Akcea, Amarin, Anthera, AstraZeneca, Boehringer Ingelheim, CSL Behring, CSL Seqirus, Daiichi Sankyo, Eli Lilly, Esperion, Merck, Omthera, Resverlogix, Sanofi-Regeneron, Scribe, Silence Therapeutics, Takeda, and Vaxxinity. Dr Bhatt has served on the Advisory Board of Angiowave, Antlia Bioscience, Bayer, Boehringer Ingelheim, CellProthera, Cereno Scientific, E-Star Biotech, High Enroll, Janssen, Level Ex, McKinsey, Medscape Cardiology, Merck, NirvaMed, Novo Nordisk, Repair Biotechnologies, Stasys, SandboxAQ (stock options), Tourmaline Bio, Viatris; has served on the Board of Directors of the American Heart Association New York City, Angiowave (stock options), Bristol Myers Squibb (stock), DRS.LINQ (stock options), High Enroll (stock); has served as a consultant for Alnylam, Altimmune, Broadview Ventures, Corcept Therapeutics, Corsera, GlaxoSmithKline, Hims, SERB, SFJ, Summa Therapeutics, Worldwide Clinical Trials; has served on Data Monitoring Committees for Acesion Pharma, Assistance Publique-Hôpitaux de Paris, Baim Institute for Clinical Research, Boston Scientific (Chair, PEITHO trial), Cleveland Clinic, Contego Medical (Chair, PERFORMANCE 2), Duke Clinical Research Institute, Mayo Clinic, Mount Sinai School of Medicine (for the ABILITY-DM trial, funded by Concept Medical; for ALLAY-HF, funded by Alleviant Medical), Novartis, Population Health Research Institute; Rutgers University (for the NIH-funded MINT Trial); has received honoraria from the American College of Cardiology (Senior Associate Editor, Clinical Trials and News, ACC.org; Chair, ACC Accreditation Oversight Committee), Arnold and Porter law firm (work related to Sanofi/Bristol Myers Squibb clopidogrel litigation), Baim Institute for Clinical Research (AEGIS-II executive committee funded by CSL Behring), Belvoir Publications (Editor in Chief, Harvard Heart Letter), Canadian Medical and Surgical Knowledge Translation Research Group (clinical trial steering committees), CSL Behring (AHA lecture), Duke Clinical Research Institute, Engage Health Media, HMP Global (Editor in Chief, Journal of Invasive Cardiology), Medtelligence/ReachMD (CME steering committees), MJH Life Sciences, Oakstone CME (Course Director, Comprehensive Review of Interventional Cardiology), Philips (Becker's Webinar on AI), Population Health Research Institute, WebMD (CME steering committees), Wiley (steering committee); other: Clinical Cardiology (Deputy Editor, unpaid); Progress in Cardiovascular Diseases (Deputy Editor, unpaid); Added Health (Editorial Board; stock options); has patent for Sotagliflozin (named on a patent for sotagliflozin assigned to Brigham and Women's Hospital who assigned to Lexicon; neither I nor Brigham and Women's Hospital receive any income from this patent); has received research funding from Abbott, Acesion Pharma, Afimmune, Alnylam, Amarin, Amgen, AstraZeneca, Atricure, Bayer, Boehringer Ingelheim, Boston Scientific, CellProthera, Cereno Scientific, Chiesi, Cleerly, CSL Behring, Faraday Pharmaceuticals, Fractyl, Idorsia, Janssen, Javelin, Lexicon, Lilly, Medtronic, Merck, MiRUS, Moderna, Novartis, Novo Nordisk, Pfizer, PhaseBio, Regeneron, Reid Hoffman Foundation, Roche, Sanofi, Stasys, 89Bio; has received royalties from Elsevier (Editor, Braunwald’s Heart Disease); and has served as site co-investigator for Cleerly. Dr Leiter has received research support from, speaking honoraria, and/or acting as an advisor to Abbott, Amgen, AstraZeneca, Boehringer Ingelheim, Eli Lilly, GSK, HLS Therapeutics, Merck, Novartis, Novo Nordisk, and Regeneron as well as a member of a DSMB for CRISPR Therapeutics and vTv Therapeutics. Dr Mazer is supported by a Merit Award from the University of Toronto Department of Anesthesiology and Pain Medicine, and holds the Cara Phelan Chair in Critical Care at St Michael’s Hospital-Unity Health Toronto; has received advisory board honoraria/consulting fees from Amgen, Alexion, AstraZeneca, BioAge, Biotest, Boehringer Ingelheim, Cardior, CSL Behring, Cytosorbents, Delex, ONA, PhaseBio, Sandoz, Trimedic Therapeutics, and Werfen as well as data safety monitoring board stipends from Beth Israel Deaconess Medical Center, Cerus, Takeda, and VarmX. Dr Verma has received grants and/or research support and/or speaking honoraria from Amgen, AstraZeneca, Bayer, Boehringer Ingelheim, Canadian Heart Research Centre, Canadian Medical and Surgical Knowledge Translation Research Group, Eli Lilly, HLS Therapeutics, Humber River Health, Janssen, Merck, Novartis, Novo Nordisk, Pfizer, PhaseBio, S & L Solutions Event Management Inc, Sanofi, and Sun Pharma. All other authors have reported that they have no relationships relevant to the contents of this paper to disclose.

## References

[1] Gaudino M., Antoniades C., Benedetto U. (2017). Mechanisms, consequences, and prevention of coronary graft failure. Circulation.

[2] Sandner S., Antoniades C., Caliskan E. (2024). Intra-operative and post-operative management of conduits for coronary artery bypass grafting: a clinical consensus statement of the European Society of Cardiology Working Group on Cardiovascular Surgery and the European Association for Cardio-Thoracic Surgery Coronary Task Force. Eur J Cardiothorac Surg.

[3] Gaudino M., Sandner S., An K.R. (2023). Graft failure after coronary artery bypass grafting and its Association with patient characteristics and clinical events: a pooled individual patient data analysis of clinical trials with imaging follow-up. Circulation.

[4] Alboom M., Browne A., Sheth T. (2023). Conduit selection and early graft failure in coronary artery bypass surgery: a post hoc analysis of the Cardiovascular Outcomes for People Using Anticoagulation Strategies (COMPASS) coronary artery bypass grafting study. J Thorac Cardiovasc Surg.

[5] Harskamp R.E., Alexander J.H., Ferguson T.B. (2016). Frequency and predictors of internal mammary artery graft failure and subsequent clinical outcomes: insights from the project of ex-vivo vein graft engineering via transfection (PREVENT) IV trial. Circulation.

[6] Jawitz O.K., Cox M.L., Ranney D. (2020). Outcomes following revascularization with radial artery bypass grafts: insights from the PREVENT-IV trial. Am Heart J.

[7] Gaudino M., Benedetto U., Fremes S.E. (2020). Angiographic outcome of coronary artery bypass grafts: the radial artery database international alliance. Ann Thorac Surg.

[8] Achouh P., Boutekadjirt R., Toledano D. (2010). Long-term (5- to 20-year) patency of the radial artery for coronary bypass grafting. J Thorac Cardiovasc Surg.

[9] Gaudino M., Tondi P., Benedetto U. (2016). Radial artery as a coronary artery bypass conduit: 20-year results. J Am Coll Cardiol.

[10] Xenogiannis I., Zenati M., Bhatt D.L. (2021). Saphenous vein graft failure: from pathophysiology to prevention and treatment strategies. Circulation.

[11] Goodman S.G., Aylward P.E., Szarek M. (2019). Effects of alirocumab on cardiovascular events after coronary bypass surgery. J Am Coll Cardiol.

[12] Tang L., Chen H., Hu X.Q. (2023). Intensive lipid-lowering therapy as per the Latest Dyslipidemia Management Guideline in predicting favorable long-term clinical outcomes in patients undergoing coronary artery bypass grafting: a retrospective cohort study. J Am Heart Assoc.

[13] Kulik A., Abreu A.M., Boronat V., Ruel M. (2019). Intensive versus moderate statin therapy and early graft occlusion after coronary bypass surgery: the Aggressive Cholesterol Therapy to inhibit Vein Graft Events randomized clinical trial. J Thorac Cardiovasc Surg.

[14] Sabatine M.S., Giugliano R.P., Keech A.C. (2017). Evolocumab and clinical outcomes in patients with cardiovascular disease. N Engl J Med.

[15] O'Donoghue M.L., Giugliano R.P., Wiviott S.D. (2022). Long-term evolocumab in patients with established atherosclerotic cardiovascular disease. Circulation.

[16] Mazer C.D., Moody A.R., Teoh H. (2025). SLICE-CEA CardioLink-8: a randomized trial of evolocumab on carotid artery atherosclerotic plaque characteristics in asymptomatic high-risk carotid stenosis. Circulation.

[17] Nicholls S.J., Kataoka Y., Nissen S.E. (2022). Effect of evolocumab on coronary plaque phenotype and burden in statin-treated patients following myocardial infarction. JACC Cardiovasc Imaging.

[18] Nicholls S.J., Puri R., Anderson T. (2018). Effect of evolocumab on coronary plaque composition. J Am Coll Cardiol.

[19] Verma S., Leiter L.A., Teoh H. (2025). Effect of evolocumab on saphenous vein graft patency after coronary artery bypass surgery (NEWTON-CABG CardioLink-5): an international, randomised, double-blind, placebo-controlled trial. Lancet.

[20] Hammer-Hansen S., Kofoed K.F., Kelbaek H. (2009). Volumetric evaluation of coronary plaque in patients presenting with acute myocardial infarction or stable angina pectoris-a multislice computerized tomography study. Am Heart J.

[21] Mancini G.B.J., Kamimura C., Yeoh E., Ryomoto A., Mazer C.D. (2022). Measurement of plaque characteristics using coronary computed tomography angiography: achieving high interobserver performance. CJC Open.

[22] Mancini G.B.J., Kamimura C., Yeoh E., Ryomoto A. (2024). Effects of adaptive or fixed thresholds and different platforms on the assessment of plaque characteristics using coronary computed tomography angiography. J Cardiovasc Comput Tomogr.

[23] Maurovich-Horvat P., Ferencik M., Voros S., Merkely B., Hoffmann U. (2014). Comprehensive plaque assessment by coronary CT angiography. Nat Rev Cardiol.

[24] Marwan M., Taher M.A., El Meniawy K. (2011). In vivo CT detection of lipid-rich coronary artery atherosclerotic plaques using quantitative histogram analysis: a head to head comparison with IVUS. Atherosclerosis.

[25] Schlett C.L., Maurovich-Horvat P., Ferencik M. (2013). Histogram analysis of lipid-core plaques in coronary computed tomographic angiography: ex vivo validation against histology. Invest Radiol.

[26] Lee S.E., Chang H.J., Sung J.M. (2018). Effects of statins on coronary atherosclerotic plaques: the PARADIGM Study. JACC Cardiovasc Imaging.

[27] Han D., Berman D.S., Miller R.J.H. (2020). Association of cardiovascular disease risk factor burden with progression of coronary atherosclerosis assessed by serial coronary computed tomographic angiography. JAMA Netw Open.

[28] Yin P., Dou G., Yang X. (2021). Noninvasive quantitative plaque analysis identifies hemodynamically significant coronary arteries disease. J Thorac Imaging.

[29] Knatterud G.L., Rosenberg Y., Campeau L. (2000). Long-term effects on clinical outcomes of aggressive lowering of low-density lipoprotein cholesterol levels and low-dose anticoagulation in the post coronary artery bypass graft trial. Post CABG investigators. Circulation.

[30] Zhu J., Zhu Y., Zhang M. (2022). Influence of lipoproteins and antiplatelet agents on vein graft patency 1 year after coronary artery bypass grafting. J Thorac Cardiovasc Surg.

[31] Kulik A., Voisine P., Mathieu P. (2011). Statin therapy and saphenous vein graft disease after coronary bypass surgery: analysis from the CASCADE randomized trial. Ann Thorac Surg.

[32] Thangasparan S., Kamisah Y., Ugusman A., Mohamad Anuar N.N., Ibrahim N. (2024). Unravelling the mechanisms of oxidised low-density lipoprotein in cardiovascular health: current evidence from in vitro and in vivo studies. Int J Mol Sci.

[33] Sorokin V., Vickneson K., Kofidis T. (2020). Role of vascular smooth muscle cell plasticity and interactions in vessel Wall inflammation. Front Immunol.

[34] Mineo C. (2020). Lipoprotein receptor signalling in atherosclerosis. Cardiovasc Res.

[35] Szarek M., Leiter L.A., Teoh H. (2026). Relationship between achieved LDL-C and saphenous vein graft patency: a post hoc analysis of the NEWTON-CABG CardioLink-5 Study. Eur J Prev Cardiol.

[36] Komiyama N., Nakanishi S., Nishiyama S., Seki A. (1996). Intravascular imaging of serial changes of disease in saphenous vein grafts after coronary artery bypass grafting. Am Heart J.

[37] Murphy G.J., Angelini G.D. (2004). Insights into the pathogenesis of vein graft disease: lessons from intravascular ultrasound. Cardiovasc Ultrasound.

[38] Hong Y.J., Mintz G.S., Kim S.W. (2009). Disease progression in nonintervened saphenous vein graft segments a serial intravascular ultrasound analysis. J Am Coll Cardiol.

[39] Jim M.H., Hau W.K., Ko R.L. (2010). Virtual histology by intravascular ultrasound study on degenerative aortocoronary saphenous vein grafts. Heart Vessels.

[40] Lee S.Y., Kim S.-W., Hong Y.J., Doh J.H. (2022). Comparison of IVUS findings between arterial and venous grafts in patients after Coronary Artery Bypass Surgery. J Cardiovasc Interv.

[41] Steg P.G., Szarek M., Bhatt D.L. (2019). Effect of alirocumab on mortality after Acute Coronary Syndromes. Circulation.

[42] Schwartz G.G., Steg P.G., Szarek M. (2018). Alirocumab and cardiovascular outcomes after Acute Coronary Syndrome. N Engl J Med.

[43] Bohula E.A., Marston N.A., Bhatia A.K. (2026). Evolocumab in patients without a previous Myocardial Infarction or stroke. N Engl J Med.

[44] Verma S., Bhatt D.L., Steg P.G. (2021). Icosapent ethyl reduces ischemic events in patients with a history of previous coronary artery bypass grafting: reduce-it CABG. Circulation.

[45] Verma S., Mazer C.D., Fitchett D. (2018). Empagliflozin reduces cardiovascular events, mortality and renal events in participants with type 2 diabetes after coronary artery bypass graft surgery: subanalysis of the EMPA-REG OUTCOME(R) randomised trial. Diabetologia.

[46] Verma S., Emerson S., Plutzky J. (2025). Semaglutide improves cardiovascular outcomes in patients with history of coronary artery bypass graft and obesity. J Am Coll Cardiol.

[47] Kulik A., Ruel M., Jneid H. (2015). Secondary prevention after coronary artery bypass graft surgery: a scientific statement from the American Heart Association. Circulation.

[48] Paquin A., Poirier P., Beaudoin J., Piché M.E. (2020). Secondary prevention after CABG: do new agents change the paradigm?. Curr Opin Cardiol.

