# Supplementary material for: Effect of Evolocumab on Grafts Post-Coronary Artery Bypass Surgery: Post-Hoc Analysis of Newton-CABG CardioLink-5
Source: JACC Adv. 2026 Aug 19;5(9):103162. doi: 10.1016/j.jacadv.2026.103162 (PMC13520735; doi:10.1016/j.jacadv.2026.103162)
Supplement: Supplementary file 1 [file mmc1.docx]

**Supplemental Material**

Standard Operating Procedure	Page 1

Supplemental Figure 1. Consort diagram	Page 6

Supplemental Figure 2. Treatment Group Comparison of IMA Graft Focal Stenosis Severity	Page 7

Supplemental Figure 3. Treatment Group Comparison of Radial Graft Focal Stenosis Severity	Page 8

Supplemental Table 1. Demographics and baseline clinical characteristics	Page 9

Supplemental Table 2. Treatment Group Comparison of Plaque Volume by Graft Type	Page 11

Cardiovascular Imaging Research Core Laboratory

| VGH Research Pavilion | **Tel:** | (604) 875-5477 |
| --- | --- | --- |
| Rm 489 – 828 West 10^th^ Avenue | **Fax:** | (604) 875-5471 |
| Vancouver, B.C. Canada  V5Z 1L8 | **e-mail:** | [cirl@interchange.ubc.ca](mailto:cirl@interchange.ubc.ca) |

# STANDARD OPERATING PROCEDURE

SOP-Newton-CABG-04

Revision 04

## CCTA analysis

The 4th revision of this Standard Operating Procedure (SOP) has been prepared by Eunice Yeoh and is issued for use under the authority of the individuals identified below.

### Approvals:

G.B. John Mancini, MD Director,

Cardiovascular Imaging Research Core Laboratory

Date

Eunice Yeoh Chief Technician,

Cardiovascular Imaging Research Core Laboratory

Date

### Date of implementation:

This SOP has been approved by the above individuals and is effective as of	.

### References:

- SurePlaque manual for Vitrea.
- Vitrea User Guide
- Appendix A – SurePlaque Procedure for Plaque Analysis
- OM-TA001 TransferApp Operations Manual

Revision of any part of this SOP is recorded in the table below, along with a brief description of the revision. Revisions will become effective on the date that an authorized individual approves and signs the revision.

| Rev’n  No. | Revised by: | Description | Approved  by: | Signature  | Date |
| --- | --- | --- | --- | --- | --- |
| 2 | Eunice Yeoh | Page 4 - Revised definition of positive remodelling to ≥ 1.10 (as per recent publications). | Eunice Yeoh |    | 03JAN  2019 |
| 3 | Eunice Yeoh | References – added Appendix A and OM-TA001.  Pg 3 -removed baseline CT section as per protocol amendment May 2020.  Pg 4 – corrected HU thresholds for fibrous and calcified plaque  Pg 4 – revised to separate focal graft analysis from whole graft analysis Pg 4 – added definition of LAP and  spotty calcium with citations at the end Pg 5 – added section for data entry, review, and signature. | Eunice Yeoh |  | 01SEP  2021 |
| 4 | Eunice Yeoh | Pg 3 and 4– updated section 3. | Eunice Yeoh |  | 07FEB  2022 |

#### 1. Software required

- Vitrea (Vital Images)
- CIRCL TransferApp.

#### 2. Receipt and download of scans

- De-identified CCTA scans will be sent to CIRCL via the CIRCL transfer app (refer to OM-TA001 TransferApp Operations Manual for details).
- Using the CIRCL app, download the CCTA study and upload to the Vitrea workstation.
- The site will send a transmittal page with each scan.

#### 3. Analysis of Follow-Up CCTA

- Data from the analysis is entered directly into the CIRCL Access database to generate a report that is reviewed and signed by the Director.
- Vitrea Analysis steps are outlined in Appendix A – SurePlaque Procedure for Plaque Analysis.
- Screenshots of Vitrea analyses will be taken as a record of the analysis.

a) Graft analysis
   - Using the Vessel Probe tool in Vitrea, select the graft to be analyzed. Review the vessel and lumen outlines to ensure that the edge detection is accurate.

- *Focal Graft Lesion Analysis (for the worst %DS stenosis)*:
  - Ensure that the correct reference point is selected for accurate calculation of stenosis grade and remodelling index.
  - % Diameter Stenosis (%DS) is graded as a categorical variable: 0, 1-19%, 20-49%, 50-99%, or 100%. If there are multiple stenoses in a graft, the lesion with the worst

%DS is recorded.

- A focal stenosis is a plaque that has a ≥ 20 %DS.
- The remodelling index (RI) is recorded for the worst focal stenosis in each graft. SurePlaque reports the remodelling index as a percentage. Convert that to a numerical value (i.e 110% = 1.10). Indicate “yes” to positive remodelling if the remodelling index is ≥ 1.10.
  - If the stenosis grade is 0% (i.e. normal graft), remodelling index is not applicable, so no RI is entered and n/a is entered in the positive RI data field.
- Count the number of positively remodeled plaques and enter in the “Number of focal lesions with positive remodelling” data field.
- Count the number of low-attenuation plaques and enter into the “Number of LAP plaques” data field. A plaque is considered to be a low-attenuating plaque if ≥25% of the total plaque volume is LAP.^1-3^
- Visually assess for spotty calcification in the focal stenosis. Spotty calcification is defined as presence for detectable calcium ≤3mm in any direction but comprising less than 90◦ of the lesion circumference^4,^ ^5^. Count the number of lesions with spotty calcium and enter into the “Number of lesions with spotty calcium” data field.
- *Whole Graft Atheroma Analysis:*
  - Ensure that SurePlaque settings are set appropriately: low attenuation plaque (LAP) <30HU, fibrous 30-349 HU, and calcified ≥350 HU.
  - Record the volume and volume percentage for each plaque component.
  - Record the length measured in the plaque analysis.
  - The total volumes and volume percentages are calculated automatically in the Access database.

b) Native Coronary Analysis
   - The native segment(s) distal to the last graft anastomosis is visually assessed for stenosis grade
   - Enter the segment name of the native segment distal to the last anastomosis for each graft. If there is more than one coronary segment distal to a graft, each segment is assessed as a separate data field.
   - Visually grade the % diameter stenosis in each segment as 0-49, 50-99, or 100%.
c) Scan Quality
   - Grade the scan quality as good or sub-optimal.
   - Note any technical issues such as motion, heavy calcium, poor contrast, misregistration, or noise. Technical issues not included in the checklist are noted in the Other, specify field.

#### 4. Data review, data entry and signatures

d) Review analysis with another technician. If there are questions, review with the Core Lab Director (or another supervising physician).
e) Select your name as the analyzing technician and enter the date completed in the database. Print the report and forward to the Core Lab Director for review and signature.
f) Enter data into electronic capture system (<https://newton.esocdat.smh.ca/)> that is provided and maintained by the study sponsor.

#### References

1. Maurovich-Horvat P, Ferencik M, Voros S, Merkely B, Hoffmann U. Comprehensive plaque assessment by coronary CT angiography. *Nat. Rev. Cardiol.* 2014;11:390-402.
2. Marwan M, Taher MA, El Meniawy K, et al. In vivo CT detection of lipid-rich coronary artery atherosclerotic plaques using quantitative histogram analysis: a head to head comparison with IVUS. *Atherosclerosis.* 2011;215:110-115.
3. Schlett CL, Maurovich-Horvat P, Ferencik M, et al. Histogram analysis of lipid-core plaques in coronary computed tomographic angiography: ex vivo validation against histology. *Invest. Radiol.* 2013;48:646-653.
4. Han D, Berman DS, Miller RJH, et al. Association of Cardiovascular Disease Risk Factor Burden With Progression of Coronary Atherosclerosis Assessed by Serial Coronary Computed Tomographic Angiography. *JAMA Netw Open.* 2020;3:e2011444.
5. Driessen RS, de Waard GA, Stuijfzand WJ, et al. Adverse Plaque Characteristics Relate More Strongly With Hyperemic Fractional Flow Reserve and Instantaneous Wave-Free Ratio Than With Resting Instantaneous Wave-Free Ratio. *JACC Cardiovasc. Imaging.* 2020;13:746-756.

**Supplemental Figure 1. Consort diagram.**

**Supplemental Figure 2. Treatment Group Comparison of IMA Graft Focal Stenosis Severity.** Treatment OR (95% CI) =.98 (0.64, 1.50), *P* =.93 across stenosis severity categories. Numbers in parentheses are the number of grafts in each severity category over the total number of grafts evaluated.

**Supplemental Figure 3. Treatment Group Comparison of Radial Graft Focal Stenosis Severity.** Treatment OR (95% CI) = 1.10 (0.28, 4.36), *P* =.90 across stenosis severity categories. Numbers in parentheses are the number of grafts in each severity category over the total number of grafts evaluated.

**Supplemental Table 1. Demographics and baseline clinical characteristics of the analysis cohort by treatment assignment**

| **Characteristic** | **Evolocumab**  **(n=277)** | **Placebo**  **(n=273)** |
| --- | --- | --- |
| Age, years | 65 (59, 71) | 66 (59, 71) |
| Female sex | 40 (14.4) | 44 (16.1) |
| Race/Ethnicity |  |  |
| White | 197 (71.1) | 183 (67.0) |
| South Asian | 56 (20.2) | 66 (24.2) |
| East Asian | 10 (3.6) | 19 (7.0) |
| African descent | 4 (1.4) | 2 (0.7) |
| Other | 10 (3.6) | 3 (1.1) |
| BMI, kg/m^2^ | 28.5 (25.4, 30.9) | 27.8 (25.0, 31.2) |
| HbA1c, % | 6.2 (5.7, 7.2) | 6.2 (5.6, 7.0) |
| Systolic blood pressure, mmHg | 123 (111, 134) | 122 (113, 135) |
| Medical history |  |  |
| Myocardial infarction | 114 (41.2) | 125 (45.8) |
| Percutaneous coronary intervention | 49 (17.7) | 44 (16.1) |
| Peripheral artery disease | 10 (3.6) | 9 (3.3) |
| Stroke | 7 (2.5) | 4 (1.5) |
| Hypertension | 234 (84.5) | 212 (77.7) |
| Medications |  |  |
| Statin | 273 (98.6) | 271 (99.3) |
| Aspirin | 269 (97.1) | 265 (97.1) |
| P2Y12 Inhibitor | 92 (33.2) | 79 (28.9) |
| Beta blocker | 258 (93.1) | 252 (92.3) |
| Number of days from surgery to randomization | 12 (6, 17) | 12 (6, 16) |
| Status of index CABG procedure |  |  |
| Elective procedure | 196 (70.8) | 187 (68.5) |
| Urgent procedure | 75 (27.1) | 78 (28.6) |
| Emergency | 6 (2.2) | 8 (2.9) |
| CABG performed with CPB | 256 (92.4) | 263 (96.3) |
| Grafts per participant (SVG or arterial grafts) | 3 (3, 4) | 3 (3, 4) |
| Received SVG | 277 (100) | 273 (100) |
| SVG per participant | 2 (2, 3) | 2 (2, 3) |
| Received LIMA | 262 (94.6) | 253 (92.7) |
| Received RA | 20 (7.2) | 23 (8.3) |
| LDL-C, mmol/L | 1.84 (1.24, 2.83) | 1.86 (1.20, 2.79) |
| LDL-C, mg/dL | 71.0 (48.0, 109.4) | 71.7 (46.2, 107.7) |
| HDL-C, mmol/L | 0.97 (0.78, 1.19) | 1.00 (0.81, 1.24) |
| HDL-C, mg/dL | 37.5 (30.0, 45.8) | 38.7 (31.3, 47.8) |
| Non-HDL-C, mmol/L | 2.60 (1.89, 3.67) | 2.44 (1.88, 3.46) |
| Non-HDL-C, mg/dL | 100.5 (73.1, 141.9) | 94.4 (72.7, 133.8) |
| Triglyceride, mmol/L | 1.57 (1.13, 2.18) | 1.54 (1.07, 2.09) |
| Triglyceride, mg/dL | 139.1 (100.1, 193.1) | 136.4 (94.3, 185.1) |

BMI, body mass index; CABG, coronary artery bypass grafting; CPB, cardiopulmonary bypass; HbA1c, glycated haemoglobin; LIMA, left internal mammary artery; RA, radial artery; SVG, saphenous vein graft.

Values are medians (interquartile range) for continuous variables and n (%) for categorical variables. There were no significant differences between the two treatment arms.

**Supplemental Table 2. Treatment Group Comparison of Plaque Volume by Graft Type**

| Graft Type | Volume Outcome | Evolocumab  Mean (95% CI) | Placebo  Mean (95% CI) | P-value |
| --- | --- | --- | --- | --- |
| SVG |  | n = 263 | n = 257 |  |
|  | Total Plaque, mm^3^ | 414.8 (386.3 - 433.3) | 413.9 (385.0 - 442.8) | 0.97 |
|  | Absolute LAP, mm^3^ | 80.3 (74.5 - 86.2) | 77.6 (71.6 - 83.5) | 0.52 |
|  | Percent LAP, % | 9.6 (9.4 - 9.9) | 9.3 (9.0 - 9.5) | 0.05 |
|  | Absolute Mixed Plaque, mm^3^ | 334.4 (311.2 - 357.5) | 336.3 (312.9 - 359.7) | 0.91 |
|  | Percent Mixed Plaque, % | 40.3 (39.5 - 41.0) | 40.1 (39.3 - 40.9) | 0.74 |
|  | Absolute Calcified Plaque, mm^3^ | 0.065 (0.022 - 0.109) | 0.059 (0.015 - 0.103) | 0.84 |
|  | Percent Calcified Plaque, % | 0.009 (0.004 - 0.014) | 0.006 (0.001 - 0.011) | 0.40 |
| IMA |  | n = 222 | n = 217 |  |
|  | Total Plaque, mm^3^ | 198.0 (182.2 - 213.8) | 192.7 (176.7 - 208.7) | 0.64 |
|  | Absolute LAP, mm^3^ | 46.5 (42.5 - 50.6) | 44.8 (40.8 - 48.9) | 0.56 |
|  | Percent LAP, % | 10.8 (10.4 - 11.2) | 10.7 (10.3 - 11.1) | 0.62 |
|  | Absolute Mixed Plaque, mm^3^ | 151.5 (139.0 - 163.9) | 147.8 (135.3 - 160.4) | 0.69 |
|  | Percent Mixed Plaque, % | 35.3 (34.3 - 36.3) | 35.3 (34.3 - 36.3) | 0.97 |
|  | Absolute Calcified Plaque, mm^3^ | 0.011 (0.001 - 0.021) | 0.006 (0 - 0.017) | 0.55 |
|  | Percent Calcified Plaque, % | 0.002 (0 - 0004) | 0.001 (0 - 0.003) | 0.54 |
| Radial |  | n = 9 | n = 15 |  |
|  | Total Plaque, mm^3^ | 220.4 (95.6 - 345.1) | 229.4 (132.8 - 326.0) | 0.91 |
|  | Absolute LAP, mm^3^ | 42.2 (12.4 - 71.9) | 44.1 (21.0 - 67.1) | 0.92 |
|  | Percent LAP, % | 9.2 (7.5 - 11.0) | 10.3 (8.9 - 11.7) | 0.34 |
|  | Absolute Mixed Plaque, mm^3^ | 178.2 (81.0 - 275.3) | 185.4 (110.1 - 260.6) | 0.90 |
|  | Percent Mixed Plaque, % | 40.5 (37.4 - 43.6) | 44.9 (42.4 - 47.3) | 0.03 |
|  | Absolute Calcified Plaque, mm^3^ | 0 | 0 | 0 |
|  | Percent Calcified Plaque, % | 0 | 0 | N/A |

Total Plaque = absolute LAP + Mixed + Calcified Plaque, : CI = confidence interval
